# Endoscopic Ultrasound Guided Rendezvous Drainage of Biliary Obstruction Using a New Flexible 19-Gauge Fine Needle Aspiration Needle

**DOI:** 10.1155/2016/3125962

**Published:** 2016-10-16

**Authors:** Zhouwen Tang, Efehi Igbinomwanhia, Sherif Elhanafi, Mohamed O. Othman

**Affiliations:** ^1^Section of Gastroenterology and Hepatology, Department of Medicine, Baylor College of Medicine, Houston, TX, USA; ^2^School of Public Health, University of Texas Science Health Center at Houston, Houston, TX, USA; ^3^Division of Gastroenterology, Department of Medicine, University of Pennsylvania, Perelman School of Medicine, Philadelphia, PA, USA

## Abstract

*Background and Aim.* A successful endoscopic ultrasound guided rendezvous (EUS-RV) biliary drainage is dependent on accurate puncture of the bile duct and precise guide wire manipulation across the ampulla of Vater. We aim to study the feasibility of using a flexible 19-gauge fine aspiration needle in the performance of EUS-RV biliary drainage.* Method.* This is a retrospective case series of EUS-RV biliary drainage procedures at a single center. Patients who failed ERCP during the same session for benign or malignant biliary obstruction underwent EUS-RV using a flexible, nitinol covered, 19-gauge needle for biliary access and guide wire manipulation.* Result.* 24 patients underwent EUS-RV biliary drainage via extrahepatic access while 1 attempt was via intrahepatic access. The technical success rate was 80%, including 83.3% of cases via extrahepatic access. There was no significant difference in success rate of inpatient and outpatient procedures, benign or malignant indications, or type of guide wire used. Adverse events included mild pancreatitis (3 patients) and cholangitis (1 patient).* Conclusion.* A flexible 19-gauge needle for biliary access can be safe and effective when used to perform EUS-RV biliary drainage. Direct comparison between the nitinol needle and conventional metal needles in the performance of EUS guided biliary drainage is needed.

## 1. Introduction

Endoscopic retrograde cholangiopancreatography (ERCP) is a standard method for diagnostic and therapeutic intervention in many benign or malignant pancreaticobiliary conditions [1]. But every ERCP based intervention must start with successful initial cannulation of the major duodenal papilla, which is achieved approximately 90% of the time [2].

Various strategies have been developed to assist in cannulation of the common bile duct (CBD). In addition to cannulation with the standard biliary catheter or sphincterotome, biliary guide wire assisted cannulation [3], pancreatic duct guide wire assisted cannulation [4], pancreatic duct stent assisted cannulation [5], needle-knife papillotomy [6], and suprapapillary fistulotomy [7] have been described and are in clinical use [8]. Yet when access sphincterotomy fails to result in deep biliary cannulation or when the papilla is inaccessible such as in the setting of surgically altered anatomy, gastrointestinal tract obstruction, or high grade biliary obstruction, alternative methods of biliary access and drainage are required.

Radiological methods such as percutaneous transhepatic cholangiography (PTC) and surgical intervention are well described as alternative methods but suffer from high complication rates and significant morbidity [9, 10]. PTC is further limited by the cosmetic and nonphysiological sequelae of external biliary drainage [11].

Linear array endoscopic ultrasound (EUS) was developed as a diagnostic modality for biliary and pancreatic neoplasms but its use has since been extended, with the aid of Doppler capabilities, to a broad collection of interventional procedures, including EUS guided biliary drainage (EUS-BD) [12].

EUS-BD may be categorized according to access method and drainage route. The bile duct may be accessed by either the intrahepatic or extrahepatic portions, typically with a 19- or 22-gauge fine needle aspiration (FNA) needle. Intrahepatic access may be transgastric, transesophageal, or transjejunal  (in surgically altered anatomy) with typical initial puncture into the left hepatic lobe. Extrahepatic access is usually transduodenal and progresses into the CBD. Once accessed, biliary drainage methods include direct transmural or transluminal drainage, antegrade transpapillary placement of a biliary stent, or guide wire rendezvous with a duodenoscope allowing subsequent conventional ERCP management. This last method, termed EUS guided rendezvous (EUS-RV), is preferred as the initial option for EUS-BD by many practitioners because it avoids the creation of a permanent biliary-enteric fistula and complications of fistula creation including bile leak and perforation [13, 14].

Extrahepatic EUS-RV may be performed with the echoendoscope in either long or short positions. In the short position, the point of puncture is in the second portion of the duodenum with the needle pointed toward the papilla. However, this position suffers from relative echoendoscope instability. In the long position, the echoendoscope is stabilized in the duodenal bulb but the needle typically points toward the hepatic hilum due to the stiffness of conventional stainless steel FNA needles therefore hindering passage of the guide wire across the papilla [15].

Recently, Varadarajulu et al. [16] described the successful technical performance of a flexible nitinol covered FNA needle (Expect™ 19-gauge flex, Boston Scientific, Marlborough, MA, USA) for transduodenal sampling of the pancreatic head/uncinate, a process that had also suffered from technical limitations with conventional stiff stainless steel FNA needles [17, 18].

**Figure 1:**
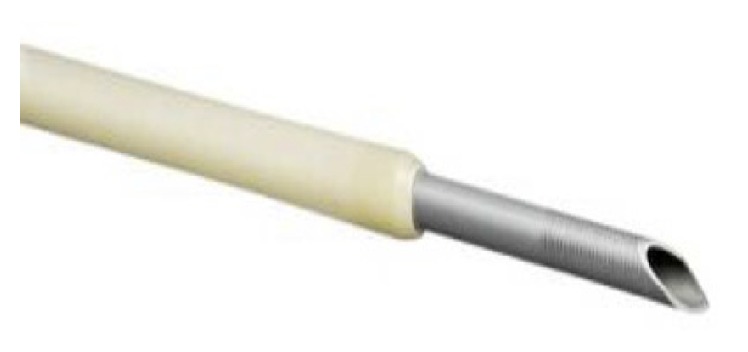
Flexible 19-gauge fine needle aspiration needle.

Experience with this flexible needle in EUS-RV has not been specifically described in the literature. Since guide wire manipulation may be a leading factor in failed EUS-BD [19], we hypothesized that using a nitinol covered flexible 19-gauge FNA needle (Figure 1) will improve the ease of performance of EUS-RV and hence serve as a viable access device. Therefore, we aim to assess the technical and clinical performance of the flexible 19-gauge FNA needle in EUS-RV.

## 2. Methods

This is a single center retrospective study of EUS guided rendezvous biliary drainage procedures. We reviewed the electronic medical and endoscopic records at University Medical Center (El Paso, TX, USA) to identify all consecutive EUS-BD procedures using the Expect 19-gauge flexible needle for biliary access from January 2012 until March 2014. Patients needing EUS-BD following failed ERCP for biliary obstruction within the same session were included. The study was approved by the Institutional Review Board of the University Medical Center at El Paso, TX, USA, and complied with Health Insurance Portability and Accountability Act regulations.

**Figure 2:**
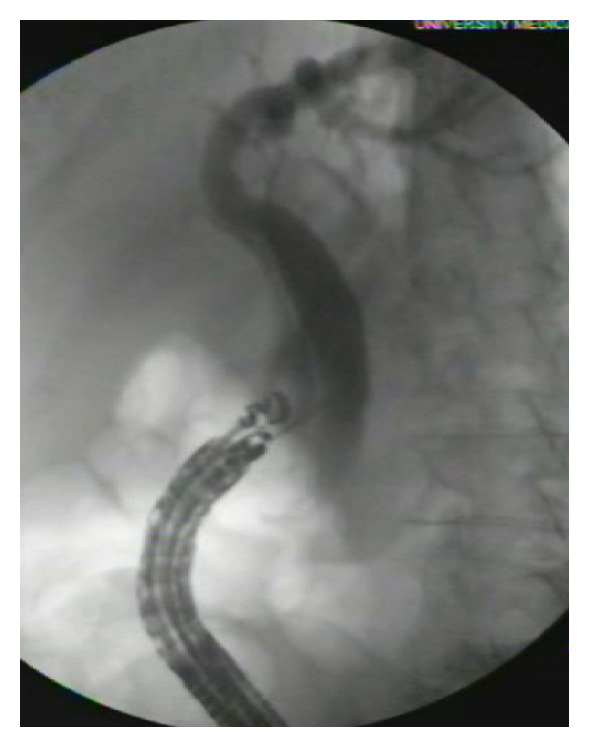
EUS guided cholangiogram.

Each procedure was performed in a hospital endoscopy unit and each patient was placed under either general anesthesia or monitored anesthesia care. Preprocedural prophylaxis antibiotics were given. A linear Olympus GF-UC140P echoendoscope (Olympus America Inc., Center Valley, PA, USA) with a ProSound SSD 5000 processor (Aloka, Wallingford, CT, USA) was used to identify the desired access point for EUS-BD. Color Doppler was used to identify intervening blood vessels. Extrahepatic versus intrahepatic access was at the discretion of the endoscopist. A 19-gauge flexible FNA needle Expect (Boston Scientific Natick, MA, USA) was used to puncture the bile duct at the desired location. Bile was aspirated and contrast medium injected for cholangiography (Figure 2). An angled tip guide wire was then inserted through the needle and passed across the ampulla of Vater into the duodenum in anterograde fashion under fluoroscopic guidance. Guide wires used were the 0.025 in × 450 cm VisiGlide (Olympus America Inc., Center Valley, PA, USA), 0.035 in × 480 cm Tracer Metro (Cook Medical, Bloomington, IN, USA), or the 0.035 in × 450 cm Dreamwire or Jagwire (Boston Scientific, Marlborough, MA, USA) per operator preference. The echoendoscope was then removed over the guide wire and a TJV-Q180V duodenoscope (Olympus America Inc., Center Valley, PA) was advanced parallel to the guide wire until it was adjacent to the ampulla. Depending on the operator preference, a standard sphincterotome was used to cannulate the CBD alongside the RV wire or the intraluminal free end of the guide wire was then grasped with forceps and brought through the working channel of the duodenoscope. After that, ERCP was performed per conventional fashion, including biliary sphincterotomy, stone extraction, stent placement, and/or stricture dilation as indicated and EUS-RV was deemed technically successful. A single expert endoscopist performed all procedures (MO).

Each patient's demographics, indications for procedure, sedation methods, procedure time, and procedural outcomes, including procedure related complications, were collected in the database. Severity of adverse events such as pancreatitis, cholangitis, or hemorrhage is defined as outlined by Schmit et al. [20]. Data is reported in descriptive statistics where applicable and categorical variables were compared using chi squared tests.

## 3. Results

Our database included 25 patients who met the inclusion criteria with mean age of 69.1 ± 16.8 years (range 22–97 years) of which 15 (60%) were females. Baseline characteristics of the study are summarized in Table 1. The indications for the procedure were malignant obstruction of the bile ducts in 13 patients (52%), benign obstruction of the bile ducts (stone/benign stricture) in 10 patients (40%), bile leak in 1 patient (4%), and biliary dilation in 1 patient (4%).

Per protocol, all 25 patients had a previously failed ERCP; reasons for failure were deformed ampulla in 8 patients (32%), failure to cannulate in 6 patients (24%), deformed duodenum in 4 patients (16%), periampullary diverticula in 4 patients (16%), and biliary stricture in 3 patients (12%).

Mean procedure time, including EUS-RV after failed conventional ERCP in a single session, was 103 ± 38 minutes. VisiGlide guide wire was used in 13 (52%) procedures with the balance of the procedures using other guide wires detailed above. Procedures using the VisiGlide guide wire had a success rate of 92.3% (12 of 13 cases) while procedures using other guide wires had a success rate of 67.85 (8 of 12 cases). This difference was not statistically significant (*P* = 0.11). There was also no statistically significant difference in success rates between cases performed for benign (10/12, 83.3%) versus malignant indications (10/13, 77%, *P* = 0.69) or between inpatients (13/18, 72%) and outpatients (7/7, 100%, *P* = 0.11).

Successful EUS guided biliary access and cholangiogram were achieved in all the 25 patients. Successful EUS-RV was achieved in 20 of 25 patients (80%), including 20 of 24 patients (83.3%) in which extrahepatic biliary access was attempted (Table 2). The impossibility to guide the wire into the direction of the distal common bile duct was the reason for failure in the above-mentioned four cases. All procedures were done in the long scope position.

One unsuccessful attempt at EUS-RV via intrahepatic access was made in a patient with a malignant biliary obstruction of the proximal common bile duct. In this case, puncture of the intrahepatic bile duct was successful but the guide wire could not be advanced past the common bile duct stricture and the patient was referred for PTC. This was also the only patient who had a postsurgical anatomy as she had a roux-en-y choledochojejunostomy for prior bile duct injury. The remaining patients all had normal bowel continuity.

Of the 4 patients with failed EUS-RV via extrahepatic access, 1 patient had a severe duodenal deformity of benign etiology which precluded identification of the ampulla, 1 patient had a severe duodenal deformity arising from a large head of the pancreas adenocarcinoma that prevented passage of the guide wire, 1 patient had a large cystic neoplasm in the head of the pancreas that prevented passage of the guide wire, and 1 patient had a large periampullary diverticulum. All 4 of these patients were referred for PTC.

Complications were reported in 4 (16%) patients. Three patients (12%) had mild acute pancreatitis. One patient had cholangitis (4%). All complications were resolved within 3 days after the procedure. There was no periprocedural mortality.

## 4. Discussion

Interventional EUS is one of the leading fronts of endoscopic innovation and EUS-BD is an apt example of the potential for innovative, less costly, and less invasive endoscopic alternatives to traditional management techniques for high mortality gastrointestinal illness. From its first description in 1996 by Wiersema et al. [21] of EUS guided transluminal cholangiography, this technique has been advanced to direct EUS guided transluminal biliary drainage, which was first described by Giovannini et al. in 2001 [22], and subsequently to EUS-RV by Mallery et al. [23]. The specific role of EUS guided transmural drainage and EUS-RV, both with respect to each other and to non-EUS guided drainage modalities, remains to be clarified. EUS-RV seems to perform favorably compared to EUS guided transmural drainage, with retrospective studies having shown no difference in technical success or adverse event rates [13].

Our retrospective study demonstrated the efficacy and safety of a nitinol covered 19-gauge flexible FNA needle in the performance of EUS-RV. Khashab et al. [13] reported on the use of the Expect 19-gauge flex needle in a retrospective study protocol but did not specify the proportion of procedures using this needle versus conventional 22-gauge FNA needles in attempted EUS-RV. To our knowledge, the current study is the first to specifically report the performance of this flexible needle for EUS-RV.

The current study included patients with both benign and malignant indications for biliary intervention who had prior failed ERCP for a multiple reasons. We achieved successful EUS guided biliary access in 100% of patients and completed rendezvous with subsequent completion ERCP in 84% of our patients. Though we only had one patient who underwent intrahepatic biliary access, the focus on extrahepatic access was consistent with the anticipated advantage of the more flexible needle in allowing both long and short echoendoscope positioning. The flexibility of the needle allowed for 100% success in gaining biliary access and obtaining cholangiogram. Our complication rate was 16% with 3 of the 4 instances being mild acute pancreatitis. These results are consistent with a review of previous published moderate and large scale retrospective studies of EUS-RV (Table 3).

Though there has not been a published prospective study of EUS-RV methods, a randomized controlled trial comparing EUS guided hepaticogastrostomy (EUS-HPG, intrahepatic) versus EUS guided choledochoduodenstomy (EUS-CD, extrahepatic) in patients with malignant biliary stricture enrolled 9 patients who had unsuccessful EUS-RV out of 30 cases attempted in total, yielding an EUS-RV success rate of 70% (21/30). No statistically significant differences were found between the two techniques, with technical success rate of 96% for EUS-HPG and 91% for EUS-CD [31]. This is in contrast to a previously published retrospective study in which intrahepatic access route was an independent risk factor for increased complications from EUS-BD [32].

Other retrospective studies have shown no difference in complication rates between EUS-RV and EUS guided transluminal biliary drainage [13]. When compared with a historical cohort of patients who had precut papillotomy, EUS-RV resulted in better technical success without increase in complications [33].

As seen in recent study protocols, EUS-RV is starting to occupy a rational spot along the escalation of biliary access techniques between precut papillotomy and EUS guided direct transluminal biliary drainage [13, 31]. Direct transluminal drainage has been shown to be equivalent to PTC in success and complication rates when utilized after failed EUS-RV [26] and EUS-BD appears to be equivalent to ERCP for short term outcomes in relief of malignant distal CBD obstruction [32].

Our study has several limitations. It was a retrospective study at a single institution of procedures performed by a single endoscopist and therefore subject to selection bias and limiting generalizability. In addition, we did not directly compare the flexible 19-gauge FNA needle with conventional access needles used in EUS-BD. We also used different types of angle tipped guide wires in our series though technical success rates did not differ between wire types. Finally, our study included only short term periprocedural follow-up. Even so, our study fills an important need for published experience with refined EUS-BD techniques and devices in line with other recent reports [34, 35].

As with development of therapeutic ERCP a generation ago, EUS-BD will benefit from further studies to identify its place within the armamentarium of gastrointestinal interventions [36]. Because of the relative rarity of failed ERCP cannulation, future randomized trials may necessarily be multicentered. For example, EUS guided antegrade transpapillary drainage has not been directly compared with transmural drainage; such a study may be important since EUS-RV is not feasible if the papilla cannot be reached due to surgically altered anatomy or luminal obstruction. With further refinement of technique, formalization of protocol, and development of dedicated devices, EUS-BD may yet become an effective solution to the knotty problem of biliary drainage after failed ERCP.

## Figures and Tables

**Table 1:** Baseline characteristics of study population.

Characteristics	EUS-RV (*n* = 25)
Age, y, mean (±SD)	69.1 (±16.8)
Female, number (%)	15 (60)
*Race*	
Hispanic, number (%)	15 (60)
White, number (%)	8 (32)
Other, number (%)	2 (8)
BMI (±SD)	27.4 (±6.5)
*Indications*	
Malignant obstruction, number (%)	13 (52)
Benign obstruction, number (%)	10 (40)
Bile leak, number (%)	1 (4)
Biliary dilation, number (%)	1 (4)
Inpatient, number (%)	18 (72)
Outpatient, number (%)	7 (28)

**Table 2:** Clinical outcomes.

	Success, number (%)	*P* value
Overall	20/25 (80)	
Extrahepatic access	20/24 (83.3)	
Intrahepatic access	0/1 (0)	
Benign Malignant	10/12 (83.3) 10/13 (77)	0.69
Inpatient Outpatient	13/18 (72) 7/7 (100)	0.11
VisiGlide guidewire Other guidewires	12/13 8/12	0.11

**Table 3:** Outcomes of EUS-RV Studies > 15 Patients; a: 5 patients with failed intrahepatic access subsequently underwent extrahepatic access.

Study	Method	Needle size	Extrahepatic access	Intrahepatic access	Overall	Adverse events	Adverse events details
			Success rate, % (*n*)	Success rate, % (*n*)	Success rate, % (*n*)	Rate, % (*n*)	
Kahaleh et al. [24] (2006)	RS	19- or 22-gauge	70 (7/10)	61 (11/18)	78 (18/23)^†^	17 (4/23)	Pneumoperitoneum 2, bile leak 1, bleeding 1
Shah et al. [25] (2012)	RS	19- or 22-gauge	NS	NS	74 (37/50)	12 (6/50)	Pancreatitis 4, bile leak 1, duodenal perforation 1
Artifon et al. [26] (2012)	RS	19-gauge	98 (57/58)	NS	98 (57/58)	3 (2/58)	Pericholedochal contrast leak 2
Iwashita et al. [27] (2012)	RS	19-gauge	81 (25/31)	44 (4/9)	73 (29/40)	13 (5/40)	Pancreatitis 2, abdominal pain 1, pneumoperitoneum 1, sepsis/death within four days of procedure 1
Park et al. [28] (2013)	PS	19-gauge	93 (13/14)^†^	50 (3/6)	80 (16/20)	14 (2/14)	Pancreatitis 1, bile peritonitis 1
Dhir et al. [29] (2013)	RS	19-gauge	94 (16/17)	100 (18/18)	97 (34/35)	23 (8/35)	Pain alone 4, bile leak 2, pneumoperitoneum 2
Dhir et al. [30] (2014)	RS	19-gauge	NS	NS	100 (20/20)	15 (3/20)	NS
Current study	RS	19-gauge	83.3 (20/24)	0 (0/1)	80 (20/25)	16 (4/25)	Pancreatitis 3, cholangitis 1

^†^5 patients with failed intrahepatic access subsequently underwent successful extrahepatic access.

NS, not specified; RS, retrospective; PS, prospective.

## Additional Points

Successful completion of EUS guided rendezvous biliary drainage is dependent on accurate puncture of the bile duct and precise guide wire manipulation across the ampulla of Vater. A flexible nitinol covered 19-gauge needle for biliary access can be safe and effective when used to perform EUS guided rendezvous biliary drainage.
